# Supplementary material for: bModelTest: Bayesian phylogenetic site model averaging and model comparison
Source: BMC Evol Biol. 2017 Feb 6;17:42. doi: 10.1186/s12862-017-0890-6 (PMC5294809; doi:10.1186/s12862-017-0890-6)
Supplement: Additional file 1 — Appendix. (PDF 459 kb) [file 12862_2017_890_MOESM1_ESM.pdf]

Appendix

list of all transition/transversion split models

model number	r_{ac}	r_{ag}	r_{at}	r_{cg}	r_{ct}	r_{gt}	name
0	1	1	1	1	1	1	JC69/F81
1	1	2	1	1	2	1	K80/HKY
2	1	2	1	1	2	3	
3	1	2	1	1	3	1	TN93
4	1	2	1	1	3	4	
5	1	2	1	3	2	1	
6	1	2	1	3	2	3	
7	1	2	1	3	2	4	
8	1	2	1	3	4	1	
9	1	2	1	3	4	3	
10	1	2	1	3	4	5	
11	1	2	3	1	2	1	
12	1	2	3	1	2	3	
13	1	2	3	1	2	4	
14	1	2	3	1	4	1	
15	1	2	3	1	4	3	
16	1	2	3	1	4	5	
17	1	2	3	3	2	1	K81
18	1	2	3	3	2	3	
19	1	2	3	3	2	4	
20	1	2	3	3	4	1	TIM
21	1	2	3	3	4	3	
22	1	2	3	3	4	5	
23	1	2	3	4	2	1	
24	1	2	3	4	2	3	
25	1	2	3	4	2	4	
26	1	2	3	4	2	5	TVM
27	1	2	3	4	5	1	
28	1	2	3	4	5	3	
29	1	2	3	4	5	4	
30	1	2	3	4	5	6	GTR

List of all named models, and potential models to split into

JC69/F81 111111 : 121121

K80/HKY 121121 : 121131, 123321

TN93 121131 : 123341

K81 123321 : 123341,123324

TIM 123341 : 123345

123324 : 123345,123425

123345 : 123456

TVM 123425 : 123456

123345 : 123456

GTR 123456 :

List of all transition/tranversionsplit models, and potential models to split into

111111 : 121121
121121 : 121123, 121131, 121321, 121323, 123121, 123123, 123321, 123323
121123 : 121134, 121324, 123124, 123324
121131 : 121134, 121341, 121343, 123141, 123143, 123341, 123343
121134 : 121345, 123145, 123345
121321 : 121324, 121341, 123421, 123423
121323 : 121324, 121343, 123424
121324 : 121345, 123425
121341 : 121345, 123451, 123453
121343 : 121345, 123454
121345 : 123456
123121 : 123124, 123141, 123421, 123424
123123 : 123124, 123143, 123423
123124 : 123145, 123425
123141 : 123145, 123451, 123454
123143 : 123145, 123453
123145 : 123456
123321 : 123324, 123341, 123421
123323 : 123324, 123343, 123423, 123424
123324 : 123345, 123425
123341 : 123345, 123451
123343 : 123345, 123453, 123454
123345 : 123456
123421 : 123425, 123451
123423 : 123425, 123453
123424 : 123425, 123454
123425 : 123456
123451 : 123456
123453 : 123456
123454 : 123456
123456 :

List of all reversible models, and potential models to split into

111111 : 111112, 111121, 111122, 111211, 111212, 111221, 111222, 112111, 112112, 112121, 112122, 112211, 112212, 112221, 112222, 121111, 121112, 121121, 121122, 121211, 121212, 121221, 121222, 122111, 122112, 122121, 122122, 122211, 122212, 122221, 122222
111112 : 111123, 111213, 111223, 112113, 112123, 112213, 112223, 121113, 121123, 121213, 121223, 122113, 122123, 122213, 122223
111121 : 111123, 111231, 111232, 112131, 112132, 112231, 112232, 121131, 121132, 121231, 121232, 122131, 122132, 122231, 122232
111122 : 111123, 111233, 112133, 112233, 121133, 121233, 122133, 122233
111123 : 111234, 112134, 112234, 121134, 121234, 122134, 122234
111211 : 111213, 111231, 111233, 112311, 112312, 112321, 112322, 121311, 121312,

121321, 121322, 122311, 122312, 122321, 122322
111212 : 111213, 111232, 112313, 112323, 121313, 121323, 122313, 122323
111213 : 111234, 112314, 112324, 121314, 121324, 122314, 122324
111221 : 111223, 111231, 112331, 112332, 121331, 121332, 122331, 122332
111222 : 111223, 111232, 111233, 112333, 121333, 122333
111223 : 111234, 112334, 121334, 122334
111231 : 111234, 112341, 112342, 121341, 121342, 122341, 122342
111232 : 111234, 112343, 121343, 122343
111233 : 111234, 112344, 121344, 122344
111234 : 112345, 121345, 122345
112111 : 112113, 112131, 112133, 112311, 112313, 112331, 112333, 123111, 123112, 123121, 123122, 123211, 123212, 123221, 123222
112112 : 112113, 112132, 112312, 112332, 123113, 123123, 123213, 123223
112113 : 112134, 112314, 112334, 123114, 123124, 123214, 123224
112121 : 112123, 112131, 112321, 112323, 123131, 123132, 123231, 123232
112122 : 112123, 112132, 112133, 112322, 123133, 123233
112123 : 112134, 112324, 123134, 123234
112131 : 112134, 112341, 112343, 123141, 123142, 123241, 123242
112132 : 112134, 112342, 123143, 123243
112133 : 112134, 112344, 123144, 123244
112134 : 112345, 123145, 123245
112211 : 112213, 112231, 112233, 112311, 123311, 123312, 123321, 123322
112212 : 112213, 112232, 112312, 112313, 123313, 123323
112213 : 112234, 112314, 123314, 123324
112221 : 112223, 112231, 112321, 112331, 123331, 123332
112222 : 112223, 112232, 112233, 112322, 112323, 112332, 112333, 123333
112223 : 112234, 112324, 112334, 123334
112231 : 112234, 112341, 123341, 123342
112232 : 112234, 112342, 112343, 123343
112233 : 112234, 112344, 123344
112234 : 112345, 123345
112311 : 112314, 112341, 112344, 123411, 123412, 123421, 123422
112312 : 112314, 112342, 123413, 123423
112313 : 112314, 112343, 123414, 123424
112314 : 112345, 123415, 123425
112321 : 112324, 112341, 123431, 123432
112322 : 112324, 112342, 112344, 123433
112323 : 112324, 112343, 123434
112324 : 112345, 123435
112331 : 112334, 112341, 123441, 123442
112332 : 112334, 112342, 123443
112333 : 112334, 112343, 112344, 123444
112334 : 112345, 123445
112341 : 112345, 123451, 123452
112342 : 112345, 123453
112343 : 112345, 123454

112344 : 112345, 123455
112345 : 123456
121111 : 121113, 121131, 121133, 121311, 121313, 121331, 121333, 123111, 123113, 123131, 123133, 123311, 123313, 123331, 123333
121112 : 121113, 121132, 121312, 121332, 123112, 123132, 123312, 123332
121113 : 121134, 121314, 121334, 123114, 123134, 123314, 123334
121121 : 121123, 121131, 121321, 121323, 123121, 123123, 123321, 123323
121122 : 121123, 121132, 121133, 121322, 123122, 123322
121123 : 121134, 121324, 123124, 123324
121131 : 121134, 121341, 121343, 123141, 123143, 123341, 123343
121132 : 121134, 121342, 123142, 123342
121133 : 121134, 121344, 123144, 123344
121134 : 121345, 123145, 123345
121211 : 121213, 121231, 121233, 121311, 123211, 123213, 123231, 123233
121212 : 121213, 121232, 121312, 121313, 123212, 123232
121213 : 121234, 121314, 123214, 123234
121221 : 121223, 121231, 121321, 121331, 123221, 123223
121222 : 121223, 121232, 121233, 121322, 121323, 121332, 121333, 123222
121223 : 121234, 121324, 121334, 123224
121231 : 121234, 121341, 123241, 123243
121232 : 121234, 121342, 121343, 123242
121233 : 121234, 121344, 123244
121234 : 121345, 123245
121311 : 121314, 121341, 121344, 123411, 123413, 123431, 123433
121312 : 121314, 121342, 123412, 123432
121313 : 121314, 121343, 123414, 123434
121314 : 121345, 123415, 123435
121321 : 121324, 121341, 123421, 123423
121322 : 121324, 121342, 121344, 123422
121323 : 121324, 121343, 123424
121324 : 121345, 123425
121331 : 121334, 121341, 123441, 123443
121332 : 121334, 121342, 123442
121333 : 121334, 121343, 121344, 123444
121334 : 121345, 123445
121341 : 121345, 123451, 123453
121342 : 121345, 123452
121343 : 121345, 123454
121344 : 121345, 123455
121345 : 123456
122111 : 122113, 122131, 122133, 122311, 122313, 122331, 122333, 123111
122112 : 122113, 122132, 122312, 122332, 123112, 123113
122113 : 122134, 122314, 122334, 123114
122121 : 122123, 122131, 122321, 122323, 123121, 123131
122122 : 122123, 122132, 122133, 122322, 123122, 123123, 123132, 123133
122123 : 122134, 122324, 123124, 123134

122131 : 122134, 122341, 122343, 123141
122132 : 122134, 122342, 123142, 123143
122133 : 122134, 122344, 123144
122134 : 122345, 123145
122211 : 122213, 122231, 122233, 122311, 123211, 123311
122212 : 122213, 122232, 122312, 122313, 123212, 123213, 123312, 123313
122213 : 122234, 122314, 123214, 123314
122221 : 122223, 122231, 122321, 122331, 123221, 123231, 123321, 123331
122222 : 122223, 122232, 122233, 122322, 122323, 122332, 122333, 123222, 123223, 123232, 123233, 123322, 123323, 123332, 123333
122223 : 122234, 122324, 122334, 123224, 123234, 123324, 123334
122231 : 122234, 122341, 123241, 123341
122232 : 122234, 122342, 122343, 123242, 123243, 123342, 123343
122233 : 122234, 122344, 123244, 123344
122234 : 122345, 123245, 123345
122311 : 122314, 122341, 122344, 123411
122312 : 122314, 122342, 123412, 123413
122313 : 122314, 122343, 123414
122314 : 122345, 123415
122321 : 122324, 122341, 123421, 123431
122322 : 122324, 122342, 122344, 123422, 123423, 123432, 123433
122323 : 122324, 122343, 123424, 123434
122324 : 122345, 123425, 123435
122331 : 122334, 122341, 123441
122332 : 122334, 122342, 123442, 123443
122333 : 122334, 122343, 122344, 123444
122334 : 122345, 123445
122341 : 122345, 123451
122342 : 122345, 123452, 123453
122343 : 122345, 123454
122344 : 122345, 123455
122345 : 123456
123111 : 123114, 123141, 123144, 123411, 123414, 123441, 123444
123112 : 123114, 123142, 123412, 123442
123113 : 123114, 123143, 123413, 123443
123114 : 123145, 123415, 123445
123121 : 123124, 123141, 123421, 123424
123122 : 123124, 123142, 123144, 123422
123123 : 123124, 123143, 123423
123124 : 123145, 123425
123131 : 123134, 123141, 123431, 123434
123132 : 123134, 123142, 123432
123133 : 123134, 123143, 123144, 123433
123134 : 123145, 123435
123141 : 123145, 123451, 123454
123142 : 123145, 123452

123143 : 123145, 123453
123144 : 123145, 123455
123145 : 123456
123211 : 123214, 123241, 123244, 123411
123212 : 123214, 123242, 123412, 123414
123213 : 123214, 123243, 123413
123214 : 123245, 123415
123221 : 123224, 123241, 123421, 123441
123222 : 123224, 123242, 123244, 123422, 123424, 123442, 123444
123223 : 123224, 123243, 123423, 123443
123224 : 123245, 123425, 123445
123231 : 123234, 123241, 123431
123232 : 123234, 123242, 123432, 123434
123233 : 123234, 123243, 123244, 123433
123234 : 123245, 123435
123241 : 123245, 123451
123242 : 123245, 123452, 123454
123243 : 123245, 123453
123244 : 123245, 123455
123245 : 123456
123311 : 123314, 123341, 123344, 123411
123312 : 123314, 123342, 123412
123313 : 123314, 123343, 123413, 123414
123314 : 123345, 123415
123321 : 123324, 123341, 123421
123322 : 123324, 123342, 123344, 123422
123323 : 123324, 123343, 123423, 123424
123324 : 123345, 123425
123331 : 123334, 123341, 123431, 123441
123332 : 123334, 123342, 123432, 123442
123333 : 123334, 123343, 123344, 123433, 123434, 123443, 123444
123334 : 123345, 123435, 123445
123341 : 123345, 123451
123342 : 123345, 123452
123343 : 123345, 123453, 123454
123344 : 123345, 123455
123345 : 123456
123411 : 123415, 123451, 123455
123412 : 123415, 123452
123413 : 123415, 123453
123414 : 123415, 123454
123415 : 123456
123421 : 123425, 123451
123422 : 123425, 123452, 123455
123423 : 123425, 123453
123424 : 123425, 123454

123425 : 123456
123431 : 123435, 123451
123432 : 123435, 123452
123433 : 123435, 123453, 123455
123434 : 123435, 123454
123435 : 123456
123441 : 123445, 123451
123442 : 123445, 123452
123443 : 123445, 123453
123444 : 123445, 123454, 123455
123445 : 123456
123451 : 123456
123452 : 123456
123453 : 123456
123454 : 123456
123455 : 123456
123456 :

List of parameters reported by the model

- BMT_ModelInidicator is the index of the substitution model as listed in the Appendix.
- substmodel is the model represented as a 6-digit number, where the position of the digit refers to rates ac, ag, at, cg, ct and gt respectively, and equal digits indicates that rates are shared, so 111111 is Jukes Cantor (if frequencies are kept equal), 121121 is HKY, 123456 is GTR etc.
- rateAC,...,rateGT are the rates according to the model. ESSs should be good for these rates, but if you plot joint-marginals of pairs you may find high correlation between some of these rates.
- BMT_Rates.1 to 6 are the rates used to build up the rate matrix. If only low parameter models are samples, the higher rates will be sampled very infrequently, and you should expect low ESSs for them. Correlation between pairs of rates should be low.
- BM_gammaShape is the gamma shape parameter as it is being sampled. For parts of the chain that gamma rate heterogeneity is switched off, the parameter will not be sampled, and the trace will show periods where the parameter is stuck.
- hasGammaRates indicates whether gamma rate heterogeneity it used (1) or not used (0). The mean can be interpreted as the proportion of time that gamma rate heterogeneity is switched on.
- ActiveGammaShape is the gamma shape parameter when it is sampled, but it is zero when it it not sampled. To get the estimate of the mean of the shape parameter, divide the mean ActiveGammaShape by the mean of hasGammaRates.
- BMT_ProportionInvariable, hasInvariableSites and ActivePropInvariable are the value for proportion invariable similar to BMG_gammaShape, hasGammaRates and ActiveGammaShape respectively.

- hasEqualFreqs indicates whether equal frequencies are used and the mean can be interpreted as the proportion of time that equal frequencies is used. When empirical frequencies are used, this parameter is not reported.

95% HPD of models for Primates data

empirical freqs			estimated freqs		
posterior support	cumulative support	model	posterior support	cumulative support	model
21.12 %	21.12 %	121323	16.19%	16.19%	121121
17.48 %	38.60 %	123424	14.09%	30.27%	121123
13.71 %	52.31 %	123324	10.25%	40.53%	121323
10.13 %	62.44 %	123323	9.71%	50.24%	121131
7.82 %	70.26 %	121324	5.31%	55.55%	121134
7.74 %	78.00 %	121123	4.63%	60.18%	123323
5.48 %	83.48 %	123425	4.28%	64.46%	121321
2.87 %	86.35 %	123423	3.94%	68.40%	121324
2.69 %	89.03 %	121343	2.98%	71.38%	121343
2.24 %	91.28 %	123454	2.79%	74.17%	123324
1.50 %	92.78 %	123345	2.43%	76.60%	121341
1.38 %	94.16 %	123343	2.37%	78.97%	123123
1.27 %	95.42 %	123124	2.24%	81.21%	123343
			2.09%	83.30%	123424
			1.96%	85.26%	123321
			1.96%	87.21%	123124
			1.94%	89.16%	123121
			1.52%	90.68%	123143
			1.36%	92.03%	123341
			1.32%	93.36%	123423
			1.17%	94.52%	123141
			1.14%	95.67%	121345

Freqs	Site Model	rate coverage AC	AG	AT	CG	CT	GT	mean rate	Subst. Model coverage
equal	plain	94	94	98	93	97	96	95.3	97
equal	+G	96	94	91	97	96	98	95.3	95
equal	+I	95	95	94	92	94	93	93.8	96
equal	+G+I	95	95	96	95	94	97	95.3	95
unequal	plain	96	95	93	95	99	97	95.8	95
unequal	+G	93	97	90	92	95	96	93.8	97
unequal	+I	94	97	96	96	97	96	96.0	95
unequal	+G+I	93	92	97	91	93	96	93.7	96
Mean		94.5	94.9	94.4	93.9	95.6	96.1	94.9	95.8

Freqs	Site Model	Site Model coverage	α	p_{inv}	frequency coverage	frequency coverage A	C	G	T
equal	plain	100			100	100	100	100	100
equal	+G	98	90		100	100	100	100	100
equal	+I	94		92	100	100	100	100	100
equal	+G+I	98	92	90	100	100	100	100	100
unequal	plain	100			100	94	95	92	96
unequal	+G	95	94		100	95	95	93	94
unequal	+I	94		89	100	96	90	92	94
unequal	+G+I	97	95	95	100	96	95	97	96
Mean		97.0	92.8	91.5	100.0	97.6	96.9	96.8	97.5

Table S1: Results as in Table 1, but for 5 taxa, relaxed clock, sequence length of 10K

Freqs	Site Model	rate coverage AC	AG	AT	CG	CT	GT	mean rate	Subst. Model coverage
equal	plain	9	54	10	8	56	11	24.7	4
equal	+G	9	54	11	11	55	11	25.2	4
equal	+I	11	50	13	10	50	14	24.7	4
equal	+G+I	10	54	13	10	55	14	26.0	4
unequal	plain	7	32	10	9	30	9	16.2	4
unequal	+G	11	35	16	11	33	13	19.8	4
unequal	+I	12	37	14	12	35	15	20.8	4
unequal	+G+I	13	36	15	12	36	13	20.8	4
Mean		10.2	44.0	12.8	10.4	43.8	12.5	22.3	4.0

Freqs	Site Model	Site Model coverage	α	p_{inv}	frequency coverage	frequency coverage A	C	G	T
equal	plain	88			34	49	46	44	51
equal	+G	94	79		43	53	55	54	59
equal	+I	93		71	35	49	53	51	51
equal	+G+I	96	89	87	42	55	57	56	59
unequal	plain	84			100	31	36	33	44
unequal	+G	92	83		100	43	46	33	46
unequal	+I	91		69	100	37	40	38	46
unequal	+G+I	95	86	89	100	43	44	40	46
Mean		91.6	84.2	79.0	69.2	45.0	47.1	43.6	50.2

Table S2: Results as in Table 1, but for 5 taxa, relaxed clock, sequence length of 10K, substitution model fixed to HKY

Freqs	Site Model	rate coverage AC	AG	AT	CG	CT	GT	mean rate	Subst. Model coverage
equal	plain	96	96	95	91	96	92	94.3	2
equal	+G	97	96	91	95	96	97	95.3	2
equal	+I	96	98	94	97	95	96	96.0	2
equal	+G+I	92	91	96	97	93	94	93.8	2
unequal	plain	97	92	96	91	97	98	95.2	2
unequal	+G	95	97	92	90	94	93	93.5	2
unequal	+I	95	96	97	94	93	95	95.0	2
unequal	+G+I	92	94	95	92	96	95	94.0	2
Mean		95.0	95.0	94.5	93.4	95.0	95.0	94.6	2.0

Freqs	Site Model	Site Model coverage	α	p_{inv}	frequency coverage	frequency coverage A	C	G	T
equal	plain	100			99	100	100	100	100
equal	+G	92	88		100	100	100	100	100
equal	+I	96		97	100	100	100	100	100
equal	+G+I	98	92	89	98	100	100	100	100
unequal	plain	100			100	95	96	95	95
unequal	+G	95	95		100	95	97	93	92
unequal	+I	94		90	100	95	89	96	92
unequal	+G+I	97	89	90	100	93	94	97	96
Mean		96.5	91.0	91.5	99.6	97.2	97.0	97.6	96.9

Table S3: Results as in Table 1, but for 5 taxa, relaxed clock, sequence length of 10K, substitution model fixed to GTR

Freqs	Site Model	rate coverage AC	AG	AT	CG	CT	GT	mean rate	Subst. Model coverage
equal	plain	95	97	95	92	95	93	94.5	97
equal	+G	92	94	94	96	92	94	93.7	96
equal	+I	92	94	89	91	93	94	92.2	99
equal	+G+I	94	98	91	96	93	92	94.0	96
unequal	plain	94	94	94	93	92	95	93.7	96
unequal	+G	95	96	92	94	98	93	94.7	98
unequal	+I	93	94	93	94	94	92	93.3	96
unequal	+G+I	96	94	94	96	94	92	94.3	98
Mean		93.9	95.1	92.8	94.0	93.9	93.1	93.8	97.0

Freqs	Site Model	Site Model coverage	α	p_{inv}	frequency coverage	frequency coverage A	C	G	T
equal	plain	100			100	100	100	100	100
equal	+G	95	93		100	100	100	100	100
equal	+I	98		94	100	100	100	100	100
equal	+G+I	94	88	88	99	100	100	100	100
unequal	plain	99			100	92	94	95	96
unequal	+G	88	87		100	92	90	95	94
unequal	+I	98		94	100	95	95	99	96
unequal	+G+I	94	86	87	100	96	95	88	92
Mean		95.8	88.5	90.8	99.9	96.9	96.8	97.1	97.2

Table S4: Results as in Table 1, but for 16 taxa, relaxed clock, sequence length of 5K

Freqs	Site Model	rate coverage AC	AG	AT	CG	CT	GT	mean rate	Subst. Model coverage
equal	plain	96	95	93	94	95	91	94.0	96
equal	+G	91	94	92	95	93	93	93.0	96
equal	+I	98	91	94	95	96	96	95.0	93
equal	+G+I	97	99	96	97	96	98	97.2	97
unequal	plain	95	97	96	95	93	92	94.7	96
unequal	+G	95	95	97	95	94	95	95.2	96
unequal	+I	93	94	94	100	95	92	94.7	98
unequal	+G+I	94	93	97	98	93	96	95.2	97
Mean		94.9	94.8	94.9	96.1	94.4	94.1	94.9	96.1

Freqs	Site Model	Site Model coverage	α	p_{inv}	frequency coverage	frequency coverage A	C	G	T
equal	plain	100			97	100	100	100	100
equal	+G	92	86		96	100	100	100	100
equal	+I	95		93	94	100	99	99	100
equal	+G+I	94	93	88	94	100	100	100	100
unequal	plain	100			99	96	93	90	92
unequal	+G	92	89		98	93	96	97	97
unequal	+I	99		91	100	97	93	93	94
unequal	+G+I	95	87	84	100	94	95	95	95
Mean		95.9	88.8	89.0	97.2	97.5	97.0	96.8	97.2

Table S5: Results as in Table 1, but for 16 taxa, relaxed clock, sequence length of 1K

Freqs	Site Model	rate coverage AC	AG	AT	CG	CT	GT	mean rate	Subst. Model coverage
equal	plain	93	96	94	93	97	98	95.2	94
equal	+G	92	94	98	96	95	97	95.3	94
equal	+I	97	94	94	93	95	91	94.0	96
equal	+G+I	99	95	96	97	93	99	96.5	95
unequal	plain	90	97	90	98	93	97	94.2	96
unequal	+G	95	95	98	96	94	96	95.7	97
unequal	+I	96	95	96	95	97	93	95.3	98
unequal	+G+I	95	93	94	93	93	95	93.8	98
Mean		94.6	94.9	95.0	95.1	94.6	95.8	95.0	96.0

Freqs	Site Model	Site Model coverage	α	p_{inv}	frequency coverage	frequency coverage A	C	G	T
equal	plain	100			89	99	100	100	99
equal	+G	93	88		85	100	100	100	100
equal	+I	98		96	85	93	100	100	100
equal	+G+I	93	87	85	81	94	99	100	100
unequal	plain	98			99	95	92	90	87
unequal	+G	93	94		98	92	97	98	94
unequal	+I	99		90	99	91	93	95	93
unequal	+G+I	94	87	85	98	95	94	95	94
Mean		96.0	89.0	89.0	91.8	94.9	96.9	97.2	95.9

Table S6: Results as in Table 1, but for 16 taxa, relaxed clock, sequence length of 0.5K

	5 taxa 10K sequences				
	jModelTest				bModelTest
	AIC	AIC	BIC	BIC	
	95% coverage	best fit	95% coverage	best fit	coverage
Equal Freq	92	53	90	82	97
Equal Freq + G	85	47	84	72	96
Equal Freq + G + I	93	46	86	70	97
Equal Freq + I	93	44	87	78	96
Unequal Freq	93	42	89	75	97
Unequal Freq + G	84	47	79	69	95
Unequal Freq + G + I	87	45	77	68	93
Unequal Freq + I	88	52	81	77	97
mean:	89.4	47.0	84.1	73.9	96

	16 taxa 1K sequences				
	jModelTest				bModelTest
	AIC	AIC	BIC	BIC	
	95% coverage	best fit	95% coverage	best fit	coverage
Equal Freq	73	34	71	48	96
Equal Freq + G	76	34	74	40	96
Equal Freq + G + I	73	32	68	42	97
Equal Freq + I	72	38	74	48	93
Unequal Freq	73	43	73	45	96
Unequal Freq + G	69	37	65	38	96
Unequal Freq + G + I	62	30	59	35	97
Unequal Freq + I	70	37	72	39	98
mean:	71.0	35.6	69.5	41.9	96.1

	16 taxa 0.5K sequences				
	jModelTest				bModelTest
	AIC	AIC	BIC	BIC	
	95% coverage	best fit	95% coverage	best fit	coverage
Equal Freq	66	31	66	32	94
Equal Freq + G	73	41	69	38	94
Equal Freq + G + I	64	32	56	31	95
Equal Freq + I	64	31	63	38	96
Unequal Freq	72	37	67	34	96
Unequal Freq + G	58	28	56	29	97
Unequal Freq + G + I	58	21	55	21	98
Unequal Freq + I	59	25	57	27	98
mean:	64.3	30.8	61.1	31.3	96

Table S7: Model coverage by jModelTest and bModelTest. **jModelTest 95% coverage** columns show the number of times the true substitution model was included in the smallest set of substitution models covering 95% of the weight according to the AIC and BIC criterion in jModelTest. **jModelTest best fit** columns show the number of times the best scoring model according to the BIC criterion is the true model. **bModelTest coverage** columns show the number of times the 95% credible set of substitution models contains the true model.
